# Proteomic Study Identifies Glycolytic and Inflammation Pathways Involved in Recurrent Otitis Media

**DOI:** 10.3390/ijms21239291

**Published:** 2020-12-05

**Authors:** Blendi Ura, Fulvio Celsi, Luisa Zupin, Giorgio Arrigoni, Ilaria Battisti, Bartolomea Gaita, Domenico Leonardo Grasso, Eva Orzan, Raffaella Sagredini, Egidio Barbi, Sergio Crovella

**Affiliations:** 1 Institute for Maternal and Child Health–IRCCS “Burlo Garofolo”, 65/1 Via dell’Istria, 34137 Trieste, Italy; blendi.ura@burlo.trieste.it (B.U.); luisa.zupin@burlo.trieste.it (L.Z.); mea.gaita.mg@gmail.com (B.G.); domenicoleonardo.grasso@burlo.trieste.it (D.L.G.); eva.orzan@burlo.trieste.it (E.O.); raffaella.sagredini@burlo.trieste.it (R.S.); egidio.barbi@burlo.trieste.it (E.B.); 2 Department of Biomedical Sciences, University of Padova, Via U. Bassi 58/B, 35121 Padova, Italy; giorgio.arrigoni@unipd.it (G.A.); ilaria.battisti@studenti.unipd.it (I.B.); 3 Proteomics Center, University of Padova and Azienda Ospedaliera di Padova, Via G. Orus 2/B, 35129 Padova, Italy; 4 CRIBI Biotechnology Center, University of Padova, Via U. Bassi 58/B, 35121 Padova, Italy; 5 Department of Medical, Surgery and Health Sciences, University of Trieste, 34149 Trieste, Italy; 6 Department of Biological and Environmental Sciences, College of Arts and Sciences, Qatar University—Women′s College of Sciences Building, Doha 2713, Qatar; crovelser@gmail.com

**Keywords:** recurrent otitis, adenotonsillar hypertrophy, 2-DE, proteomics

## Abstract

Recurrent acute otitis media (RAOM) in children is clinically defined as the occurrence of at least three episodes of acute otitis media over a course of 6 months. A further common pathological condition of interest in the context of pediatric otolaryngology is adenotonsillar hypertrophy (ATH), a common cause of obstructive sleep apnea syndrome. Aimed at unraveling the differential modulation of proteins in the two pathologies and at understanding the possible pathways involved in their onset, we analyzed the proteomic profile of the adenoids from 14 RAOM and ATH patients by using two-dimensional gel electrophoresis (2-DE) and mass spectrometry (MS). The 2-DE coupled with MS allowed us to identify 23 spots with significant (*p*-value < 0.05) changes in protein amount, recognizing proteins involved in neutrophil degranulation and glycolysis pathways.

## 1. Introduction

Recurrent acute otitis media (RAOM) is a disorder in which a child experiences at least three episodes of acute otitis media over a course of 6 months or four episodes across 12 months [1]. A key symptom of acute otitis media is ear pain, which might be difficult to evaluate in nonverbal children; other symptoms may include fever, irritability, otorrhea, anorexia, and sometimes vomiting or lethargy [2]. The percentage of RAOM occurrence in children under 7 years of age has been estimated to be between 20% and 30% [3]; the quality of life of both children and parents is significantly affected, especially for the social limitations due to the disease. Furthermore, parents are concerned about hearing loss, language impairment and the cognitive impact of RAOM, even if after the age of 2 a significant improvement of quality of life and pathology is expected [4].

The clinical treatment of RAOM is complex and different strategies could be employed to reduce the frequency of the episodes, including mainly oral antibiotic treatment [1] and adenotonsillectomy. Unfortunately, both have limited efficacy on the recurrence of episodes [4].

Adenoidal tissue is also involved in adenotonsillar hypertrophy (ATH), another taxing pathology for the pediatric population; ATH is characterized by an enlargement of mucosal tissue, with consequent obstructive sleep apnoea syndrome (OSAS) causing a sleep disorder with episodes of partial or complete obstruction of upper airway tract during sleep, followed by transient rousing that restores normal airway functionality [5]. The impact of OSAS on children’s quality of life is also meaningful, ranging from significant sleep disturbance to daytime sleepiness and irritability up to cardiovascular and neurological complications, growth disorders and enuresis in the most severe cases [6].

ATH etiology is poorly defined; the main hypothesis points principally towards an allergic response that can provoke enlargement of adenoidal tissue [7,8]. A further link is provided by a recent research reporting an increased level of interleukin (IL)-17A in ATH patients [9]; this cytokine appears to be involved in the development of severe asthma [10], thus further indicating a possible role for allergic response in ATH development. Therapeutic possibilities for ATH include the use of intranasal corticosteroids [11] and surgical removal of the enlarged tissue to improve respiration during sleep [12].

Aiming to assess the etiopathogenetic mechanisms of different diseases in the pediatric field, some of the so-called “omics” technologies as genomics [13], transcriptomics [14], proteomics [15] and metabolomics [16] have been used.

In the context of RAOM, different, comprehensive tools have been employed in the attempt to unravel the disease’s pathogenesis; considering that genetic predisposition has been shown to be a relevant risk factor in RAOM [17], various genetic studies have been performed to identify the genes associated with susceptibility to this pathology, recently reviewed by Giese et al. [18]. In this work, the authors associated the development of RAOM with dysfunction in different mechanisms including immune response, bacterial adhesion and viral infection. A transcriptome analysis of ear exudate from patients with RAOM highlighted the involvement of inflammation and hypoxia response in the development of a specific complication of the disease, i.e., ear effusion [19]. Moreover, a proteomic analysis of ear effusions in RAOM patients, demonstrated the involvement of neutrophils’ response, specifically neutrophil extracellular traps [20].

Similarly, a proteomic approach has been used to determine possible biomarkers for OSAS in blood and urine, identifying the proteins involved in oxidative stress and lipid metabolism [21].

These studies showed the usefulness of the “omic” approach to clarify the pathological mechanisms involved in development of RAOM. Aimed at assessing the different proteins modified in RAOM and ATH, in the attempt to consider the molecular pathways involved in both diseases, we focused on a proteomic analysis in the adenoidal tissue, after surgical removal.

Previously, a proteomic study has been performed by Just et al.; the authors analyzed the tonsillar tissue in children with chronic tonsillitis and ATH with 2 Dimensional- gels (2D-gels) followed by matrix-assisted laser desorption/ionization mass spectrometry (MALDI-MS) protein identification, reporting an increased abundance of two proteins, Heat-Shock Protein-27 (HSP27) and Uridine monophosphate (UMP)/cytidine monophosphate (CMP) kinase (UMP-CMP kinase), specifically in tissue from chronic tonsillitis patients, suggesting a possible alteration of the kinase activity and thus changes in metabolic activity of this tissue [22].

In analogy with the above mentioned work, we analyzed the proteomic profile of adenoidal tissues from RAOM and ATH, by using two-dimensional electrophoresis (2-DE) followed by mass spectrometry (MS) (2-Dimensional Electrophoresis -Liquid Chromatography -Mass Spectrometry/Mass Spectrometry, in short 2-DE-LC-MS/MS). Our goal was the identification of differentially modulated proteins in adenoidal tissue from RAOM and ATH patients.

## 2. Results

### 2.1. Proteomics

**Figure 1:**
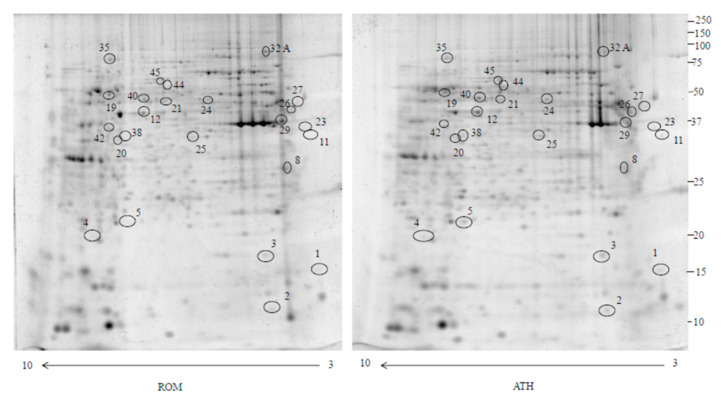
Two dimensional electrophoresis map of the adenotonsillar hypertrophy (ATH) and recurrent acute otitis media (RAOM) proteome. Immobilized pH gradient pH 3–10 non-linear strips were used for the first dimension and 12% polyacrylamide gels were used for the second dimension. Number correspond to different proteins identified in Table 1.

In this study, we used 2-DE and MS analysis to compare the proteomic profiles of adenoidal tissue from RAOM and ATH patients. Analysis was repeated for seven sets of patients, RAOM and ATH, obtaining comparable results between each pair: an average matching efficiency of approximately 80%, and a median of 2000 spots for each gel were detected. Figure 1 shows an example of a gel pair (RAOM and ATH). Image and statistical analyses indicated that 6 protein spots were significantly more intense (>1.5-fold) while 17 were significantly less intense (<0.6-fold) (Table 1) in RAOM patients compared to ATH. Fold changes were calculated as the ratio of the mean percentage volume (%V = Volume single spot/Volume total spots) of each spot between RAOM and ATH. The 23 protein spots were subjected to in-gel digestion and LC-MS/MS analysis. Proteins identified by searching the MS/MS data against the human section of the UniProt database (as described in Materials and Methods section) are listed in Table 1.

### 2.2. Identification of Pathways Involved in RAOM and ATH

**Figure 2:**
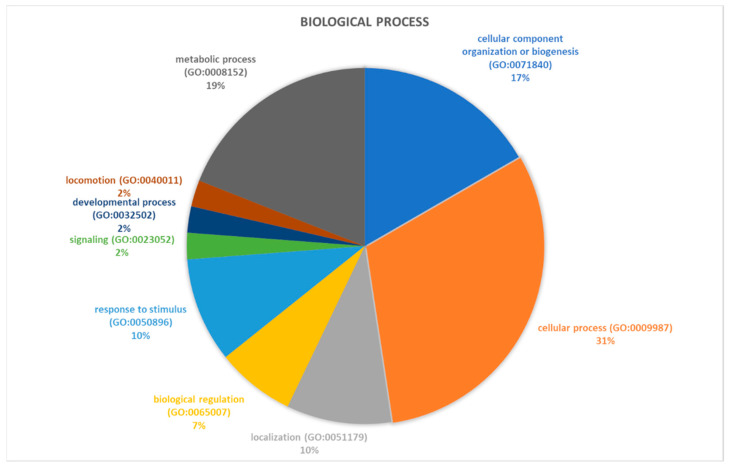
PANTHER classification of differently regulated proteins in RAOM in according to their biological processes.

**Figure 3:**
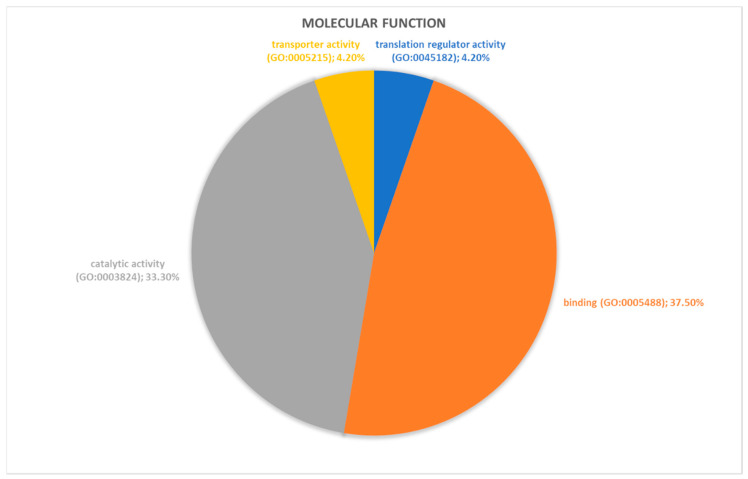
PANTHER classification of differently regulated proteins in RAOM in according to their molecular function.

**Figure 4:**
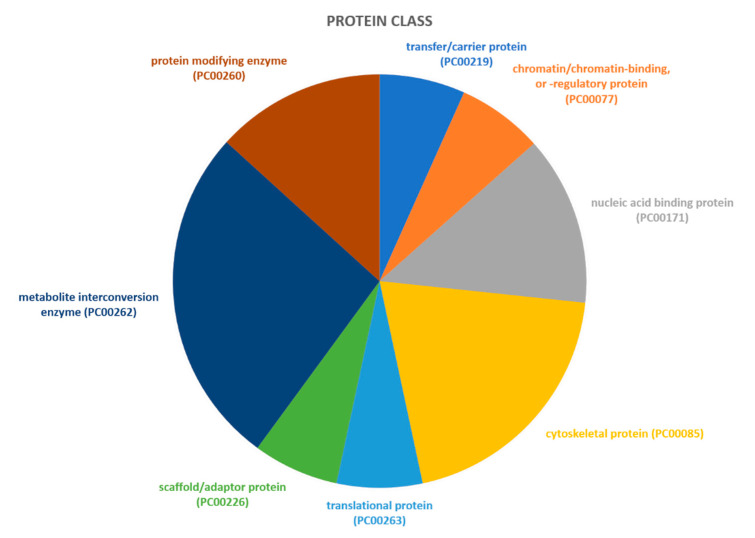
PANTHER classification of differently regulated proteins in RAOM in accordance to their protein class.

**Figure 5:**
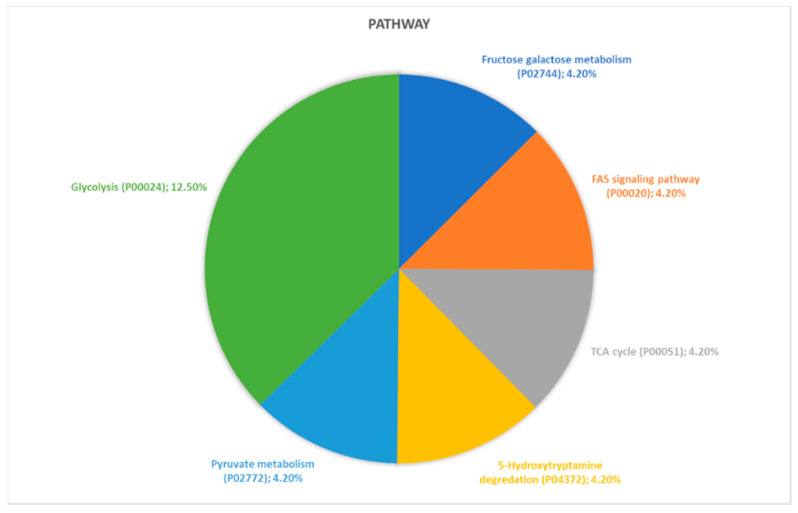
PANTHER classification of differently regulated proteins in RAOM in according to their pathway classification.

Proteins identified through proteomic analysis were then subjected to Protein Analysis Through Evolutionary Relationships (PANTHER) classification, which categorized these proteins into groups according to their biological processes, molecular function, protein class and pathway. In terms of biological processes (Figure 2), the proteins were grouped into four main categories: metabolic processes, cellular processes, cellular component organization or biogenesis, and localization. Cellular process was the category including the majority of proteins (twelve, specifically Aconitase 2 (ACO2), Albumin (ALB), Aldolase, Fructose-Bisphosphate C (ALDOC), Cyclase Associated Actin Cytoskeleton Regulatory Protein 1 (CAP1), Capping Actin Protein, Gelsolin Like (CAPG), Coronin 1A (CORO1A), Eukaryotic Translation Elongation Factor 1 Alpha 1 (EEF1A1), H4 Clustered Histone 1 (H4C1), Hemoglobin Subunit Beta (HBB), Pyruvate Kinase M1/2 (PKM), Proteasome 26S Subunit ATPase 6 (PSMC6), Valosin Containing Protein (VCP)) found in proteomic analysis. For the molecular function category (Figure 3), proteins were grouped into: catalytic activity, binding, transporter activity, and translation regulator activity, with most proteins pertaining to the binding group. For the protein class category (Figure 4), proteins were grouped in: cytoskeletal protein, metabolite interconversion enzyme, nucleic acid binding protein, and protein-modifying enzyme. Finally, regarding pathway classification, proteins were grouped into six pathways (Figure 5). Five of these (5-Hydroxytryptamine degradation, Fructose galactose metabolism, Glycolysis, Pyruvate metabolism, TCA cycle) are correlated with sugar, acid citric, and serotonin metabolism. 

Aiming to better elucidate cellular pathways altered by the differential modulated proteins we performed an enrichment analysis using DAVID based on the KEGG database. Table 2 displays the significant pathways found using this database, being hsa00010: Glycolysis/Gluconeogenesis the one with lower FDR and in common with PANTHER classification. To further expand our analysis, we performed the same enrichment analysis on another database, the REACTOME. Table 3 displays the 10 most enriched pathways; the most significant pathways are Neutrophil degranulation and Glycolysis, the latter in common with PANTHER classification and DAVID analysis.

Indeed, we found that proteins involved in the glycolytic cycle and pathways linked to cellular metabolism were present in the three databases examined, strengthening the indication about involvement of a change in cellular energy control in ROAM and ATH. Interestingly, we also found that in the KEGG database, a pathway linked to neutrophils degradation, the biosynthesis of antibiotics (Table 2); neutrophils do synthetize in granules microbicidal molecules, released upon degranulation in extracellular space [23]. This observation reinforces once more the involvement of neutrophils and their antimicrobial properties in ROAM and ATH.

### 2.3. Western Blot Study of Differentially Modulated Proteins

**Figure 6:**
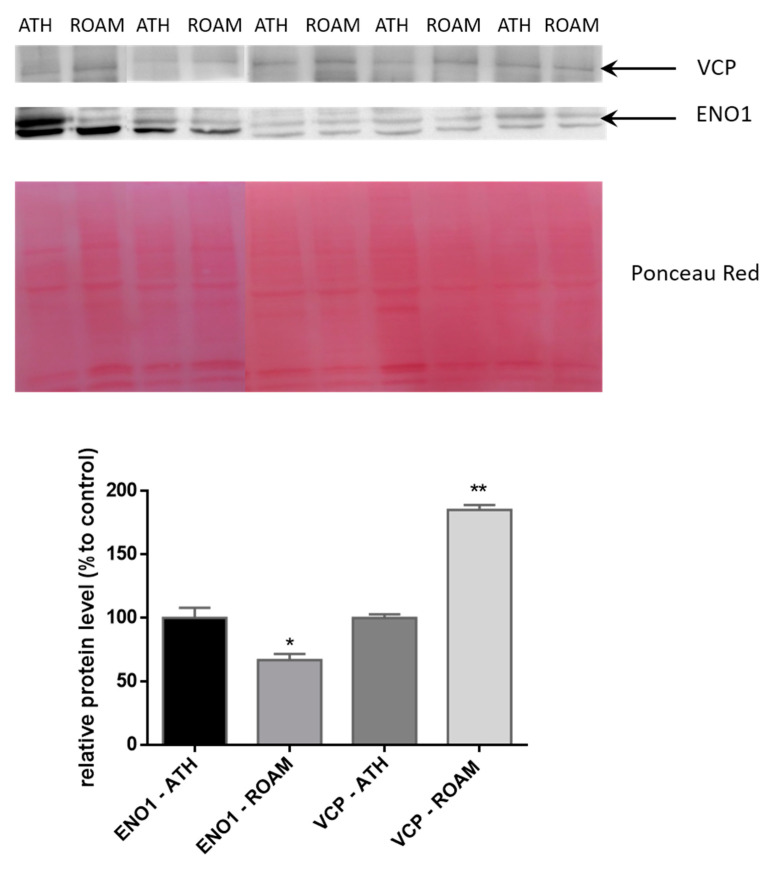
Representative Western blotting analysis of VCP and ENO1 in ATH and RAOM. The intensities of the immunostained bands were normalized with the protein intensities measured by Red Ponceau from the same blot. The bar graph shows the relative quantitation (band density) of VCP and ENO1 in ATH and RAOM. Results are shown as a histogram (* indicates *p* < 0.05, while ** indicates *p* < 0.01 statistical difference) and each bar represents mean ± standard error.

Based on the pathways analysis we selected two different proteins to validate 2-DE and MS results: ENO1 and VCP. ENO1, showing a reduced abundance (0.63 fold) in ROAM (Table 1) with respect to ATH, is involved in glycolysis and is modulated at a medium level in tonsils [24]. On the other hand, VCP, with an increased abundance (2.45 fold) in ROAM (Table 1) versus ATH, is involved in proteasome metabolism and is modulated at a medium-high level in tonsil [24]. Quantitative Western blotting analysis confirmed the results obtained by 2-DE data for these two proteins in RAOM and ATH tissue samples (Figure 6). Indeed, ENO1 abundance is significantly decreased (66.9 ± 10.9% relative protein level compared to ATH, *p* < 0.05) in RAOM sufferers, compared to ATH patients. Besides, VCP protein levels increased significantly (185.3 ± 8.55% compared to ATH, *p* < 0.01) in RAOM tissue, in comparison with ATH patients.

## 3. Discussion

This study shows that different specific pathways are involved in RAOM and ATH: inflammation triggering with neutrophils activation in the former and metabolism dysregulation, specifically of the glycolytic cycle, in the latter.

The proteomic approach has been previously used to tackle some questions regarding the etiopathogenesis of RAOM. The ear effusion from patients with ROAM has been examined in several works, in order to understand the mechanism at the basis of the pathology. A recent work found the presence of antimicrobial proteins and elevated concentration of cytokines in ROAM patients with ear effusion together with higher concentrations of othopatogens [25]. Previously, Val and co-workers characterized ear effusion in nine patients with chronic otitis media, finding 109 proteins, the majority of whom pertained to peptides recognized as released from neutrophils. They also showed immune-histological evidence of neutrophil extracellular traps (NETs), suggesting a role of these cells in pathogenesis of chronic otitis media [20]. 

Subsequently, the same group characterized the ear effusions of 57 ROAM patients reporting a higher protein content and an increase in peptides linked to the immune system and epithelial remodeling in viscous fluid compared to serous fluid [26]. 

In our experimental setting we employed a proteomic approach with the aim of determining protein patterns associated with either RAOM or ATH in adenoidal tissue. Using 2-DE coupled with mass spectrometry we identified 23 proteins whose abundance was either increased (6 proteins) or decreased (17 proteins) in RAOM compared to ATH tissues.

We are aware that a comparison between ROAM and ATH tissues with normal tissue would have been more informative to shed light on the molecular pathways involved in the disease onset and progression. Unfortunately, it is not possible to make a comparison with a “control” (i.e., without pathologies) tonsillar tissue, because to obtain it, a surgical procedure is necessary. Considering that we are focusing on pediatric patients, it is not ethically acceptable to ask them to undergo surgery only for research purposes, without any clinical benefit. For this reason we decided to compare two different pathologies excluding the “control” tissue. 

2-DE coupled with high resolution LC-MS/MS is a very powerful method for the identification and relative quantification of proteins in complex samples [27,28]. However, the combination of small/middle size IPG strips with the high resolution of very sensitive mass spectrometers might lead to the identification of several proteins in a single 2-DE spot. Therefore, discerning the protein that is actually responsible for the significant change in spot volume between different samples can indeed be problematic. For this study we based our choice on several criteria. 

At first keratins and hornerin were considered as contaminants and were not taken into account. Then we took into consideration only proteins which were identified with at least 3 unique peptides [29]. In spots 21,35,38,25,44,12,2,27,3,19,1,11,45,20,32A and 4, only 1 protein was identified with at least 3 unique peptides in addition to the contaminants (keratins and hornerin). For all other spots (8,29,23,40,26,5,42) we used the Mascot score and the sequence coverage to pinpoint the most abundant protein present in the spot. To note that for spots 29 and 23 the proteins considered to be the most abundant ones and therefore responsible for the variation in spot volume were validated with an alternative method. Indeed the altered abundance of VCP and ENO1 was validated with Western blot, which gave results comparable to those obtained by the proteomic analysis. See Supplemental Table S1.

Following that, we employed three different bioinformatic tools and databases firstly to categorize the proteins and secondly to assess in which pathways they could be involved.

The first of these tools (PANTHER) returned the “cellular process” as the category comprising the highest number of proteins. Our analysis showed an increased abundance of ACO2, CAP1, PSMC6 and VCP in RAOM tissue and an increased level of ALB, ALDOC, CAPG, CORO1A, EEF1A1, H4C1, HBB and PKM in ATH tissue. 

DAVID enrichment analysis allowed us to determine in which pathway the majority of proteins submitted for analysis are present. The most statistically significant one was the Glycolysis/Gluconeogenesis pathway formed by the series of reactions that convert glucose 6-phosphate to pyruvate in the cytosolic compartment [30]. Interestingly, this pathway is in common with the PANTHER classification system, further strengthening our results. Four proteins were present in this pathway, specifically ALDH2, ALDOC, ENO1 and PKM, all of them with an increased abundance in ATH tissue. We further validated by Western blot the increased levels of ENO1 in ATH tissues, suggesting a possible link between altered glycolytic cycle and ATH. Indeed, in OSAS patients, evidence shows that sleep apnoea lowers arterial oxygen saturation, inducing tissue hypoxia, which, in turn, increases glucose degradation through the glycolytic pathway [31]. A marked increase in serum lactate in OSAS patients is a clue of the above-described mechanism taking place, as observed by Ucar et al. [32] In 2016, Xu et al. analyzed the metabolomic profile of 120 different individuals, divided into OSAS patients and simple snorers (SS). The authors found a specific metabolic signature of increased glycolytic activity in OSAS patients vs. SS, suggesting that this signature could be used as a clinical marker of pathogenic OSAS [33]. OSAS has also been linked to metabolic diseases, such as type 2 diabetes, obesity and non-alcoholic fatty liver disease [34]. Taking these studies into account, it is then possible to hypothesize a role for glycolytic cycle dysregulation in ATH patients. Even though it remains to be determined whether this dysregulation could be an effect rather than a cause of respiratory dysfunction.

To further increase the strength of our findings, we exploited a third bioinformatic tool via the REACTOME database. We found two pathways with a statistical significant enrichment: glycolysis (R-HSA-70171) and neutrophil degranulation (R-HSA-6798695). The glycolytic pathway thus is further confirmed to be an important player in ROAM and ATH disease; in this analysis, three proteins were present, PKM, ALDOC, and ENO1, all of them with increased abundance in ATH tissue. Instead, six proteins were present in the neutrophils degranulation pathway, two of them with an increased abundance (CAP1, VCP) while four (EEF1A1, PKM, HBB and ALDOC) with decreased levels in RAOM tissue. This pathway includes proteins involved in activation of neutrophils in response to infection; upon reaching an inflammatory focus, these cells mobilize several subsets of granules, that contain different molecules: antimicrobial peptides, proteolytical proteins, cytokines, and inflammatory mediators [35]. It is then possible to hypothesize that inflammation, i.e., involvement of neutrophils activation, is persistently present and active in RAOM. As a matter of fact this has also been shown in previous work that reported in ear effusion from chronic otitis media the presence of proteins linked to the neutrophils extracellular traps (NETs) pathway [20], a mechanism strictly linked to neutrophils degranulation [36]. Remarkably, some pathogens have adapted to NETs, particularly *H. influenzae*, which can initiate NETs and tightly associate with these structures, avoiding phagocytic escape, thus resulting in chronic infection [37]. In fact, this pathogen is a leading cause of ROAM, as already reported by some studies [38,39].

**Figure 7:**
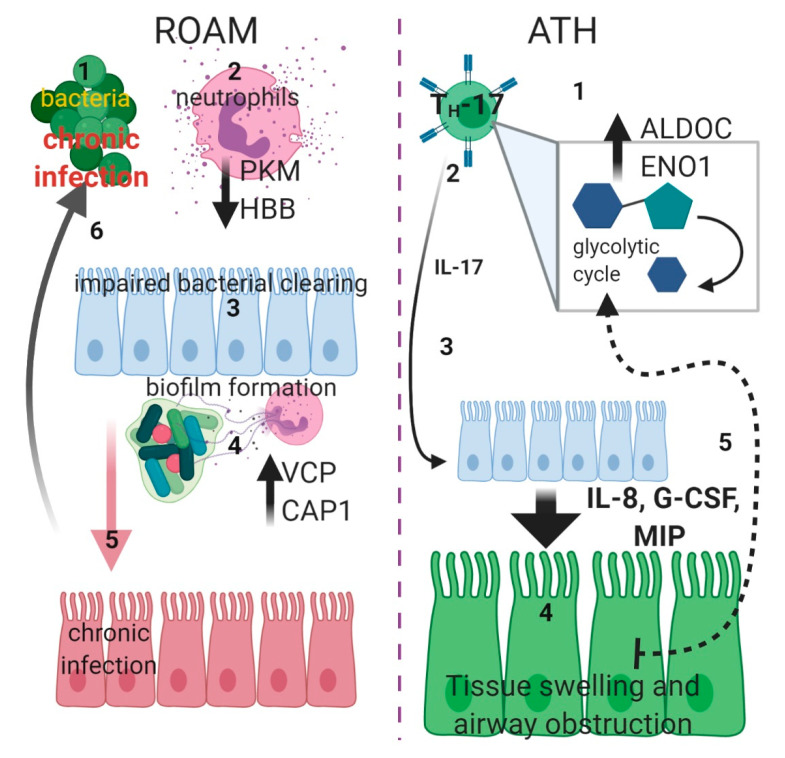
Schematic diagram displaying putative pathogenic mechanisms In ROAM (left side): bacterial infections (1) triggers neutrophils degranulation (2), but this is not effective due to lower PKM and HBB levels (black arrow downwards). Defective bacterial clearing (3) causes chronic infection (through formation of biofilm) in the median ear, with a consequent increase of NETs formation (4), due to higher proteasome activity provoked by increased VCP and CAP1 levels (black arrow upwards). Chronic bacterial infection of ear tissue (5) (red) establishes a milieu in which recurrent infections (6) are facilitated. In ATH (right side), TH-17 cells are activated (1) and switch to glycolytic metabolism (gray square), with increased levels of ALDOC and ENO1 (black arrow upwards). Activation of these cells causes increasing Interleukin-17 (IL-17) (2) production. This cytokine (3) could drive epithelial expression of granulopoietic and chemotactic factors such as Interleukin-8 (IL-8), Granulocyte Colony-Stimulating Factor (G-CSF) and Macrophage Inflammatory Proteins (MIP) (big arrow downwards) that could induce swelling of adenoidal tissue (4) (green), followed by upper airway obstruction. The imperfect oxygen intake could then trigger a self-sustained cycle (5) (dotted arrow), inducing furthermore a switch toward glycolytic metabolism (created with BioRender.com).

In conclusion, it appears that RAOM and ATH could be differentiated according to their pathogenetic mechanisms: in RAOM, chronic inflammation activation and specifically neutrophils activation seems to be predominant, while in ATH metabolism dysregulation and specifically glycolytic cycle appears to be involved in the genesis of this disease. Specifically, in ROAM patients, the decreased abundance of PKM and HBB could affect neutrophils degranulation, dampening response to infections, while the increased level of VCP and CAP1, through augmented proteasome activity, might promote the formation of NETs [40] followed by biofilm generation (as in the case of *H. influenzae*) and colonization of median ear cavity [41]. Conversely, in ATH patients, we observed an increase in glycolytic pathway-related proteins (as ALDOC or ENO1); this could represent a switch towards activation of IL-17-producing cells (TH17 cells) [42] as levels of this cytokine are elevated in ATH patients [9]. Proliferation of these cells could induce an enlargement of adenoidal tissue, followed by upper airway obstruction, which in turn increases glycolytic metabolism, triggering a self-reinforcement cycle (Figure 7).

The present study provides some new insights into the pathological mechanisms of RAOM and ATH, as we hypothesize a different trigger for each pathology: in RAOM, a failed response toward pathogenic infection, while in ATH an increase in glycolytic cycle which could in turn activate TH17 cells. These results represent the initial step to unravel the complexity of these diseases and to investigate the aetiology of both RAOM and ATH. 

A better understanding of the different pathological pathways could contribute to the design of targeted treatments, since the medical resolution of both these conditions remains at the moment elusive beside waiting years for spontaneous resolution in RAOM or recurring to surgery in ATH. 

## 4. Materials and Methods 

### 4.1. Population Characteristics

A total of 14 patients, European Caucasian, were examined (median age 8, range 4–14 years; 8 males and 6 females), 7 affected by adenotonsillar hypertrophy (ATH) and 7 presenting recurrent otitis media (RAOM). ATH patients were defined as showing an abnormal growth of pharyngeal or palatine tonsils [43], with associated OSAS as previously defined [44], with no recurrent tonsils infections (chronic tonsillitis). RAOM patients were characterized by having at least three episodes of acute otitis media (AOM) in a period of 6 months or four AOM in 12 months [1]. In these patients, surgical removal of adenotonsillar tissue was recommended as treatment for RAOM. Written informed consent for participating in the study was provided by the children’s parents. All study experiments and procedures were performed following the ethical standards of the 1975 Declaration of Helsinki (7th revision, 2013). The IRCCS “Burlo Garofolo” (Trieste, Italy) Internal Review Board (RC02/20, protocol number 02/20) and the Regional Ethic Committee (protocol number: CEUR-2020-Sper-065, approval date 6 September 2020) approved the study.

### 4.2. 2-DE and Image Analysis

Adenoidal tissue from RAOM and ATH patients was used for proteomic analysis. In brief, samples of RAOM and ATH specimens (300 mg each), washed from blood, were manually homogenized in 1.5 mL of dissolution TUC buffer (7 M urea, 2 M thiourea, 4% CHAPS, 40 mM Tris, 65 mM DTT and 0.24% Bio-Lyte (3–10)) with a protease inhibitor mix (2 mM Phenylmethylsulfonyl fluoride (PMSF), 1 mM benzamidine, 1 mM Ethylenediaminetetraacetic acid (EDTA), 1 mM Sodium fluoride (NaF)). The tissue solutions were then centrifuged at 10,000× *g* at 4 °C for 30 min and the supernatant protein content was determined using the Bradford assay. For 2-DE analysis, 300 μg of proteins from each sample were used. ReadyStrip™ 3–10 NL 17-cm immobilized pH gradient (IPG) strips were rehydrated in a dissolution buffer at 50 V for 12 h at 20 °C, and isoelectric focusing (IEF) was performed in a PROTEAN IEF Cell (Bio-Rad Laboratories, Inc., Hercules, CA, USA). After the IEF, serial incubations were performed: first, the IPG strips were equilibrated for 10 min in an equilibration buffer (6 M urea, 2% SDS, 50 mM Tris-HCl (pH 8.8), 30% glycerol) and then for 15 min in another equilibration buffer containing 4% iodoacetamide. For the second dimension, the equilibrated IPG strips were transferred to a 12% polyacrylamide gel (18.5 cm × 20 cm). After electrophoresis, gels were fixed in 40% methanol and 10% acetic acid overnight, and then stained for 6 h with Flamingo stain; 2-DE gels were scanned with a Molecular Imager PharosFX System. Double experimental replicates were performed per sample. For all gels, molecular weights were determined by comparison with Precision Plus Protein Pre-stained Standards (Bio-Rad Laboratories, Inc., Hercules, CA, USA), covering a range from 10 to 250 kDa and analyzed using the Proteomweaver 4.0 software (both from Bio-Rad Laboratories, Inc., Hercules, CA, USA) [42].

### 4.3. Quantification of Spot Levels

2-DE image analysis was performed using the Proteomweaver 4.0 software. The analysis process was carried out by matching all gels from seven RAOM and seven ATH. The Proteomweaver 4.0 algorithm matched all of the gels to find quantitative differences. Differences were considered significant when the ratio of the mean percentage relative volume (%V) (%V = V (single spot)/V (total spot)) showed a fold change of at least 1.5 and satisfied the non-parametric Wilcoxon test (*p* < 0.05). Fold change was calculated as the ratio between the mean %V of RAOM and ATH.

### 4.4. Trypsin Digestion and MS Analysis

Protein spots from 2-DE were digested and analyzed by mass spectrometry, as described by Ura et al. [45] Spots excised from 2-DE gels were washed four times with 50 mM NH_4_HCO_3_ and acetonitrile (ACN; Sigma-Aldrich, St. Louis, MO, USA) alternatively, and dried under vacuum in a SpeedVac system. For gel spot digestion, three microliters of 12.5 ng/µL sequencing grade modified trypsin (ProMega, Madison, WI, USA) in 50 mM NH_4_HCO_3_ were added, and samples were digested overnight at 37 °C. Finally, peptide extraction was performed with three changes extraction by 50% ACN/0.1% formic acid (FA; Fluka, Ammerbuch, Germany), peptide mixtures were dried under vacuum and stored at −20 °C, until mass spectrometry (MS) analysis was performed.

Samples were dissolved in 12 µL of 3% ACN/0.1% FA and 4 microliters of each sample were analyzed by LC-MS/MS with LTQ-Orbitrap XL mass spectrometer (Thermo Fisher Scientific, Waltham, MA, USA) coupled to a nano-HPLC Ultimate 3000 (Dionex—Thermo Fisher Scientific). Peptides were separated in a 10 cm pico-frit column (75 μm ID, 15 μm Tip; New Objective) packed in-house with C18 material (Aeris Peptide 3.6 µm XB-C18, Phenomenex). H_2_O/FA 0.1% and ACN/FA 0.1% were used as eluents A and B, respectively and peptides were analyzed at a flow rate of 0.25 μL/min using a linear gradient of eluent B from 3% to 40% in 20 min.

A Data Dependent Acquisition (DDA) was used: a full scan between 300 and 1700 Da was performed at high resolution (60,000) on the Orbitrap. The ten most intense ions were then selected for CID fragmentation and acquisition of MS/MS data in low resolution in the linear ion trap. Raw data files were analyzed with the software package Proteome Discoverer 1.4 (Thermo Fisher Scientific) and searched with Mascot Search Engine (version 2.2.4, Matrix Science, London, UK). Spectra were searched against the human section of the Uniprot database (version July 2018) using the following parameters: enzyme specificity was set to trypsin with 1 missed cleavage allowed, precursor and fragment ions tolerance were 10 ppm and 0.6 Da, respectively. Carbamidomethylcysteine and oxidation of methionine were set as fixed modification and variable modification, respectively. Proteins were considered as positive hits if for each protein at least 3 unique peptides were identified with high confidence (FDR < 1%). All relevant information required to assess the reliability of protein and peptide identifications is available in Supplementary Table S1. The mass spectrometry proteomics data have been deposited to the ProteomeXchange Consortium via the PRIDE [46] partner repository with the dataset identifier PXD022477.

### 4.5. Pathways Analysis

Proteins identified by MS were analyzed by the Protein Analysis Through Evolutionary Relationships (PANTHER) classification system [47]. Proteins were then classified according to their involvement in biological processes, molecular function, protein class, and pathways. To perform pathways enrichment analysis the Database for Annotation, Visualization and Integrated Discovery (DAVID) was employed on the KEGG database and further confirmed [48] and expanded using the REACTOME pathways database (https://reactome.org) aiming to better assess the pathways involved in RAOM or ATH [49]. Since the majority of the identified proteins participated in multiple processes, only the most relevant ones were reported.

### 4.6. Western Blotting

In order to validate findings obtained by proteomic analysis, we performed Western blot analysis as an orthogonal tool to confirm our observations. Proteins were chosen based on the following parameters: intermediate changes in 2-DE and mass spectrometry analysis, antibodies commercially available and participation of proteins to biological processes previously identified in bioinformatics analysis as involved in RAOM and ATH [50]. Western blotting experiments were performed as previously described [45]. Briefly, for immunoblotting analysis 30 µg of the same protein extracts used for 2-DE were separated by 12% polyacrylamide gel and then transferred to a nitrocellulose membrane. After protein transfer the membrane was blocked by treatment with 5% defatted milk in TBS-tween 20 and incubated overnight at 4 °C with 1:700 diluted primary rabbit polyclonal antibody against Transitional endoplasmic reticulum ATPase (VCP (Sigma-Aldrich; Merck KGaA, Darmstadt, Germany)), and with 1:800 diluted primary rabbit polyclonal antibody against Alpha-enolase (ENO1 (Sigma-Aldrich; Merck KGaA, Darmstadt, Germany)). After washing, membranes were incubated with HRP-conjugated anti-rabbit IgG (1:3000, Sigma-Aldrich; Merck KGaA, Darmstadt, Germany). The protein signal was visualized using SuperSignal West Pico Chemiluminescent substrate (Thermo Fisher Scientific Inc., Ottawa, ON, Canada). The intensities of the immunostained bands were normalized with the total protein intensities measured by staining the membranes from the same blot with Ponceau S solution (Sigma-Aldrich, St. Louis, MO, USA).

### 4.7. Statistical Analysis

Statistical analyses were carried out with the non-parametric Wilcoxon signed-rank test for matched samples for both 2-DE and Western blot data. *p* < 0.05 was considered to indicate a statistically significant difference. All analyses were conducted with Graph Pad Prism 5 for Windows.

## Figures and Tables

**Table 1:** Dysregulated proteins identified by mass spectrometry in RAOM compared to ATH.

Accession Number	Spot Number	Protein Description	Gene Symbol	Peptide Number	Protein Score	Fold Change *	Standard Deviation	*p*-Value
**Q5HYB6**	8	Epididymis luminal protein 189	*DKFZp686J1372*	9	558.97	6.3	±0.9	0.03
**P62333**	21	26S proteasome regulatory subunit 10B	*PSMC6*	6	202.21	3.16	±0.47	0.016
**Q01518-2**	35	Isoform 2 of adenylyl cyclase-associated protein 1	*CAP1*	11	357.54	2.6	±0.31	0.016
**P55072**	29	Transitional endoplasmic reticulum ATPase	*VCP*	9	307.23	2.45	±0.44	0.04
**P05091-2**	45	Isoform 2 of aldehyde dehydrogenase, mitochondrial	*ALDH2*	7	323.91	1.75	±0.16	0.016
**Q99798**	32 A (other spots not considered)	Aconitate hydratase, mitochondrial	*ACO2*	10	301.94	1.57	±0.28	0.016
**A0A2R8Y6G6**	23	Alpha-enolase	*ENO1*	12	755.18	0.63	±0.25	0.031
**P14618**	38	Pyruvate kinase PKM	*PKM*	14	707.25	0.62	±0.22	0.031
**P31146**	40	Coronin-1A	*CORO1A*	15	456.89	0.62	±0.13	0.032
**Q15365**	20	Poly(rC)-binding protein 1	*PCBP1*	5	200.50	0.6	±0.04	0.022
**P40121-2**	25	Isoform 2 of Macrophage-capping protein	*CAPG*	5	193.65	0.6	±0.07	0.033
**P08670**	26	Vimentin	*VIM*	15	624.65	0.52	±0.08	0.015
**P31146**	44	Coronin-1A	*CORO1A*	15	208.24	0.48	±0.11	0.032
**P68871**	12	Hemoglobin subunit beta	*HBB*	4	250.80	0.43	±0.07	0.015
**O75368**	2	SH3 domain-binding glutamic acid-rich-like protein	*SH3BGRL*	3	167.54	0.4	±0.65	0.03
**P81605**	27	Dermicidin	*DCD*	3	77.39	0.39	±0.12	0.016
**A0A087WWT3**	3	Serum albumin	*ALB*	3		0.36	±0.14	0.015
**A8MVZ9**	19	Fructose-bisphosphate aldolase	*ALDOC*	4	149.95	0.31	±0.07	0.045
**A0A0C4DG56**	5	Superoxide dismutase (Mn), mitochondrial	*SOD2*	3	125.89	0.3	±0.12	0.015
**P40121-2**	1	Isoform 2 of Macrophage-capping protein	*CAPG*	5	193.65	0.3	±0.06	0.016
**A0A087WVQ9**	4	Elongation factor 1-alpha 1	*EEF1A1*	3	90.64	0.15	±0.05	0.03
**P62805**	42	Histone H4	*H4C1*	5	210.83	0.11	±0.04	0.03
**Q01105-2**	11	Isoform 2 of Protein SET	*SET*	6	124.37	0.038	±0.01	0.03

* Fold change was defined as the ratio of the mean %Volume according to the formula %V = Volume single spot/Volume total spot of RAOM vs. ATH. *p*-value obtained using Wilcoxon test (as described in Materials and Methods).

**Table 2:** KEGG over-representation results. Count: differentially modulated proteins between RAOM and ATH found belonging to the pathway. *p*-value: The result of the Binomial Test for over-representation. FDR: False Discovery Rate, corrected over-representation probability.

Term	Count	*p*-Value	FDR
**hsa00010: Glycolysis/Gluconeogenesis**	4	2.3 × 10^−4^	6.8 × 10^−3^
**hsa01230: Biosynthesis of amino acids**	4	2.9 × 10^−4^	6.9 × 10^−3^
**hsa01130: Biosynthesis of antibiotics**	5	5.1 × 10^−4^	7.8 × 10^−3^
**hsa01200: Carbon metabolism**	4	0.001	0.013

**Table 3:** REACTOME over-representation results. Entities Found: differentially modulated proteins between RAOM and ATH found belonging to the pathway/total proteins belonging to the pathway. Ratio: numerical ratio between differentially modulated proteins between RAOM and ATH found belonging to the pathway/total proteins belonging to the pathway. *p*-value: The result of the Binomial Test for over-representation. FDR: False Discovery Rate, Corrected over-representation probability.

Pathway Name	Entities Found	Ratio	*p*-Value	FDR
**Neutrophil degranulation**	7/480	0.023	9.40 × 10^−5^	0.06
**Glycolysis**	4/124	0.006	1.80 × 10^−4^	0.06
**Glucose metabolism**	4/197	0.009	0.001	0.226
**Hh mutants that don′t undergo** **autocatalytic processing are** **degraded by ERAD**	2/63	0.003	0.009	0.402
**Hh mutants abrogate ligand secretion**	2/67	0.003	0.01	0.402
**Chaperone Mediated Autophagy**	3/200	0.01	0.01	0.402
**HSF1 activation**	2/79	0.004	0.014	0.402
**Role of ABL in ROBO-SLIT signaling**	1/10	4.78 × 10^−4^	0.022	0.402
**Late endosomal microautophagy**	2/109	0.005	0.025	0.402
**Post-translational protein phosphorylation**	2/109	0.005	0.025	0.402

## Supplementary Materials

Supplementary Materials can be found at https://www.mdpi.com/1422-0067/21/23/9291/s1.

## Abbreviations

RAOM	Recurrent Acute Otitis Media
ATH	adenotonsillar hypertrophy
2-DE	two-dimensional gel electrophoresis
MS	mass spectrometry
OSAS	obstructive sleep apnoea syndrome
IL-17A	interleukin-17A
